# The role of PPARγ in TBBPA-mediated endocrine disrupting effects in human choriocarcinoma JEG-3 cells

**DOI:** 10.1007/s11010-015-2514-z

**Published:** 2015-08-08

**Authors:** Ewelina Honkisz, Anna K. Wójtowicz

**Affiliations:** Department of Animal Biotechnology, University of Agriculture, Rędzina 1B, 30-248 Kraków, Poland

**Keywords:** Tetrabromobisphenol A, Peroxisome proliferator-activated receptor gamma, β-hCG, Progesterone, Caspase-3, JEG-3

## Abstract

The goal of the present study was to investigate the action of TBBPA on PPARγ protein expression in vitro in human choriocarcinoma-derived placental JEG-3 cells. We also analyzed TBBPA for its action on placental secretion of progesterone and β-hCG, cell viability, and apoptosis. Our results showed that after TBBPA treatment at 10 nM and 10 µM, PPARγ protein expression increased in a time-dependent manner until 48 h and then slightly decreased at 72 h but was still above the control level. This alteration in PPARγ protein expression was accompanied by a decreased β-hCG level. Interestingly, co-treatment with the PPARγ antagonist GW9662 reversed the TBBPA-mediated changes in PPARγ protein expression but, according to β-hCG secretion, potentiated an inhibitory effect of TBBPA. Additionally, in our study, we assessed the ability of TBBPA to increase progesterone levels in JEG-3 cells compared with those of controls. Finally, in the present study, we demonstrated that TBBPA at all of the tested doses significantly increased caspase-3 activity compared with that of the vehicle control. The apoptotic action of TBBPA was also confirmed by Hoechst 33342 staining. These results showed the up-regulation of PPARγ protein expression after TBBPA exposure in human placental cells. Although co-treatment with antagonist of PPARγ reversed the TBBPA-mediated increase in this protein expression and restored it to the control level, it did not reverse the effect on β-hCG secretion. This indicated that the mechanism of TBBPA-induced changes in β-hCG secretion is PPARγ-independent.

## Introduction

Tetrabromobisphenol A (TBBPA) is the highest selling brominated flame retardant and has become a hazardous environmental contaminant worldwide [1]. TBBPA is released into the environment primarily through various waste streams that are generated during manufacturing and processing and upon disposal [2], but it also leaks out from a variety of commercial products in our houses and circulates as dust [3, 4]. This contamination represents a potential to cause harm to human health. The number of studies demonstrate endocrine disrupting effects for TBBPA in both in vitro and in vivo tests [5–10].

The highly lipophilic character of this substance and, thus, its ease of crossing cell membranes should be a matter of great concern. Ogunbayo et al. [11] demonstrated the ability of TBBPA to interact with phospholipid membranes, restricting their motion, which in turn could modulate some cellular processes. Numerous epidemiological studies have shown the presence of TBBPA in body fluids, such as human blood [12–16] and breast milk [17–20]. Assuming a mean TBBPA concentration in breast milk of 0.073 ng/g fresh weight and an average daily intake of milk by a breastfed newborn of 500 ml, the intake of TBBPA can be estimated at 40 ng [17]. TBBPA has also been detected in umbilical cords [17, 20–22] at concentrations that are relevant to this study. Furthermore, the TBBPA concentration was 2–5 times higher in the infants than in the mothers [23], indicating maternal transport as a critical factor for the exposure of a developing fetus to TBBPA.

The presence of TBBPA in placenta tissue poses a serious threat to the proper functions of the organ acting as an endocrine gland, which consequently generates a risk for pregnancy health and proper development of the fetus. The human placenta is a highly specific endocrine gland that is responsible for the synthesis of a number of hormones and proteins that are necessary for normal fetal development and pregnancy maintenance. Barak et al. [24] demonstrated that peroxisome proliferator-activated receptor γ (PPARγ) null mutant placentas exhibit vascular anomalies with a failure in vascular labyrinth formation. This experimental approach revealed a key role of PPARγ in placenta development. Since then, extensive studies have considered PPAR’s control of placental biology. In the human placenta, PPARγ regulates trophoblast differentiation and invasion which are essential for fetal development and pregnancy maintenance [25–27]. Recent data indicate that TBBPA can activate PPARγ by acting as an agonist in NIH3T3-L1 preadipocytes [28]. However, there is no information available regarding the mechanism of PPARγ activation by TBBPA in the placenta cells and the potential consequences of the process. Thus, the aim of the present study was to investigate the capacity of TBBPA to interact with and perturb signaling by PPARγ. Thus the aim of the present study was to investigate the effect of TBBPA on placental hCG and progesterone secretion and signaling by PPARγ in human choriocarcinoma placental cells.

## Methods

### Cell culture

The human choriocarcinoma-derived placental JEG-3 cell line was obtained from the American Type Culture Collection (Rockville, MD, USA). The cells were maintained in DMEM without phenol red and were supplemented with 10 % charcoal-stripped FBS (and thus depleted of steroid hormones), 100 UI/ml penicillin, and 100 µg/ml streptomycin in 75-cm^2^ cell culture flasks (Nunc, Denmark). JEG-3 cells were cultured at 37 °C in a humidified atmosphere of 5 % CO_2_/95 % air. A confluent JEG-3 cell culture was detached from the flask with 0.25 % trypsin–EDTA and allowed to adhere in culture plates for at least 24 h before treatment.

### Cell exposure to chemicals

The cells were seeded in 96-well culture plates (Costar, St. Louis, USA) at a density of 8 × 10^3^ (for the 24-h treatment), 7 × 10^3^ (for the 48-h treatment) or 6 × 10^3^ (for the 72-h treatment) and initially cultured for 24 h. Subsequently, in one experiment, the medium was changed to DMEM that was supplemented with 5 % charcoal-stripped FBS in the presence of TBBPA (1 nM, 10 nM, 50 nM, 100 nM, 1 μM, 10 μM, 50 μM, or 100 μM). In a second experiment, the cells were pretreated with PPARγ agonist GW1929 (10 µM) or the PPARγ antagonist GW9662 (10 µM) for 2 h prior to the addition of TBBPA (10 nM) at the indicated time. GW1929 and GW9662 at this concentration did not alter cell viability (data not shown). For hormone analysis, the culture medium was collected after 48 h. Each stock solution of TBBPA, GW1929, and GW9662 was made in DMSO as a 1000-fold stock solution and stored at 4 °C (TBBPA) or at −20 °C (GW1929 and GW9662). For cell exposure, the incubation media were prepared through a 1000-fold dilution of the DMSO stock solution with the culture medium (0.1 % vol/vol DMSO) immediately prior to cell treating. At this concentration, DMSO had no effect on steroid secretion or cell viability. The viability of the cells was determined before seeding by the Trypan blue exclusion test and was found to be ≥95 % (data not shown). The control group was cultured in medium with 0.1 % DMSO.

After an appropriate culture time, 100 µl of medium was collected to test for cell viability, and the rest was frozen at −20 °C for hormone (progesterone and β-hCG) analyses. The cells were frozen at −80 °C and used for the estimation of apoptosis (caspase-3 activity) as described below. Every treatment was conducted in ten wells, and each experiment was repeated three times.

### Viability assay

The cytotoxic potential of TBBPA was determined by the cytotoxicity detection kit (Roche Applied Science, Germany) according to the manufacturer’s instructions. The assay is based on the detection of lactate dehydrogenase (LDH) that is released from dead cells as a result of cytotoxicity. Briefly, after 24, 48, and 72 h of exposure to increasing doses of TBBPA, 100 μl of the culture medium was transferred to 96-well microtiter plates, and the LDH activity was determined by the addition of the substrate solution. The formation of formazan was measured at 492 nm in a microplate reader (Bio-Tek ELx800). The data were analyzed with KCJunior (Bio-Tek Instruments) and were normalized to the absorbance in vehicle-treated cells. The results were expressed as the mean absorbance from thirty separate samples ± SEM. The extinction values of the control cells were set as 100 %, and the rate of LDH release from treated cells was calculated as a percentage of the control.

### β-hCG and progesterone secretion

The levels of placental hormones were measured to determine the response curve to different TBBPA concentrations during various periods of exposure. The amounts of β-hCG and progesterone were determined in the media by an enzyme-linked immunosorbent assay (ELISA) using commercial kits (DRG, Marburg, Germany) according to the manufacturer’s instructions. For β-hCG, the sensitivity of the assay was <1 mlU/ml, and the intra- and inter-assay CV values were 4 and 7.3 %, respectively. For progesterone, the sensitivity of the assay was 0.045 ng/ml, and the intra- and inter-assay CV values were 6.99 and 4.34 %, respectively. The sample concentrations were calculated using a best-fit four-parameter logistic calibration curve (KCJunior; Bio-Tek). Each treatment was analyzed in ten wells, and each experiment was repeated three times.

### Protein isolation and Western blot analysis

Protein isolation and Western blot analysis were performed as described previously [29]. The membranes were incubated overnight at 4 °C with the mouse monoclonal anti-PPARγ (sc-7273; Santa Cruz Biotechnology Inc., Santa Cruz, CA, USA) that was diluted at 1:100 in TBS/Tween. After incubation with the primary antibody, the membranes were washed with TBST (0.02 M TBS and 0.02 % Tween 20) and incubated for 1 h with goat anti-mouse HRP-conjugated antibody (sc-2005; Santa Cruz Biotechnology Inc., Santa Cruz, CA, USA) that was diluted at 1:500 in TBS/Tween. The signals were detected by chemiluminescence (ECL) using a Western Blotting Luminol Reagent (sc-2048, Santa Cruz Biotechnology Inc., Santa Cruz, CA, USA) and imaged using GeneSnap software and a G:Box Imaging system. To confirm that the protein loading was the same across the gel, the Western blot membrane was cut into strips and probed with an anti-β-actin antibody as a loading control (Sigma Chemical Co., St. Louis, MO, USA).

### Caspase-3 activity

Caspase-3 activity was used as a marker for cell apoptosis and was determined according to Nicholson et al. [30]. The enzyme activity was measured with the AC-DEVD-pNA colorimetric assay as previously described [31]. Briefly, cultured cells were lysed on ice with a lysis buffer (50 nM HEPES, pH 7.4, 100 nM NaCl, 0.1 % CHAPS, 1 nM EDTA, 10 % glycerol, and 10 nM DTT). The reaction was initiated by the addition of caspase-3 substrate Ac-DEVD-pNA (*N*-acetyl-Asp-Glu-Val-Asp-*p*-nitroanilide; Sigma) to a final concentration of 200 µM. The samples were incubated in the dark at 37 °C for 1 h. Then, the colorimetric release of *p*-nitroaniline from the Ac-DEVD-pNA substrate was recorded every 30 min and monitored continuously for 120 min at 405 nm in a microplate reader (Bio-Tek ELx800). Cells that were treated with 1 nM staurosporine were used as a positive control (Sigma, St Louis, MO, USA). The data were analyzed with KCJunior (Bio-Tek Instruments), normalized to the absorbance in vehicle-treated cells, and expressed as the percent of control ± SEM of ten separate samples that were run in triplicate. The absorbance of the blanks, acting as no-enzyme controls, was subtracted from each value. Because the initial substrate concentration was subsaturating (200 µM), only the data within the linear range of the reaction curve provided a consistent measure of caspase-3 activity.

### Identification of apoptotic cells

Apoptotic cells exhibit nuclear condensation and DNA fragmentation that can be detected by “vital” staining with Hoechst 33342 (Sigma, St Louis, MO, USA). For this purpose, JEG-3 cells were seeded on coverslips that were set in 24-well plates at a density of 8 × 10^4^/well and initially cultured for 24 h to allow for attachment. The next day, the medium was changed to DMEM that was supplemented with 5 % charcoal-stripped FBS with the addition of 10 nM or 10 µM of TBBPA, and the cells were cultured for another 24 h. After this period, the cells were washed with PBS and exposed to Hoechst 33342. Hoechst 33342 was diluted with PBS and added to the medium to a final concentration of 10 μM. The cells were incubated for 15 min in an atmosphere of 5 % CO_2_/95 % air at 37 °C and then studied with a fluorescence microscope (Nikon, Japan).

### Statistical analysis

The data were presented as the mean ± SEM of three independent experiments. Each treatment was repeated three times in ten wells. Thus, the total number of replicates was 30. The average of the repeats was used for statistical calculations. Data were analyzed by one-way analysis of variance (ANOVA). To determine the significant differences, the means were compared between the treated groups and the control group using Dunnett’s test for multiple comparisons. Significance was indicated as follows: *P < 0.05; **P < 0.01; and ***P < 0.001 (versus control cultures).

## Results

### Progesterone secretion

**Fig. 1:**
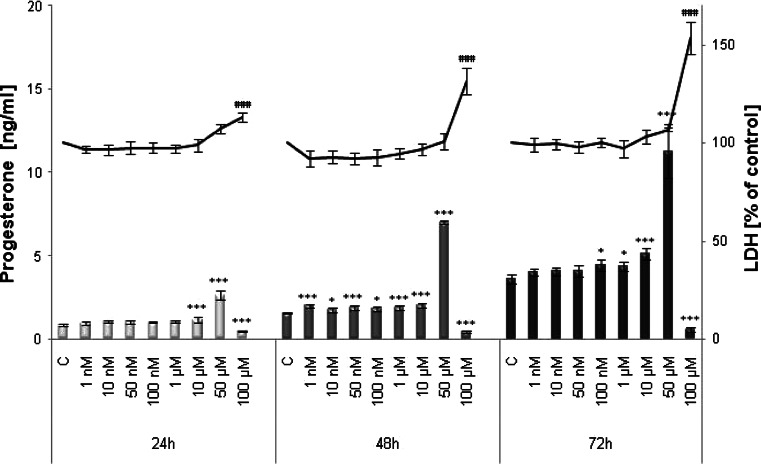
Time- and dose-dependent effect of increasing concentrations of TBBPA on JEG-3 cells viability (*line graph*) and progesterone secretion (*bar graph*). Each point represents the mean ± SEM of three independent experiments, each of which consisted of ten replicates per treatment group. Data indicated with *P < 0.05;***p < 0.001 (progesterone secretion) and ^###^ *p* < 0.001 (LDH release) reflects statistically significant differences between control and experimental groups

Increasing concentrations of TBBPA in the cultures of JEG-3 cells affected progesterone secretion (Fig. 1). TBBPA treatment for 24 h led to an increase in progesterone levels but was only significant in the micromolar range (10 and 50 µM). However, 48 h of treatment with both nanomolar and micromolar doses resulted in significant increases, with progesterone secretion ranging from approximately 12–30 %. TBBPA treatment with 50 µM seemed to be the most effective at increasing progesterone levels by 2- to 3-fold compared with those of the vehicle control (DMSO) at all of the time points. TBBPA at a concentration of 100 µM decreased progesterone secretion due to the dominant cytotoxic effect (up to 53 % LDH leakage).

### Caspase-3 activity and Hoechst 33342 staining

**Fig. 2:**
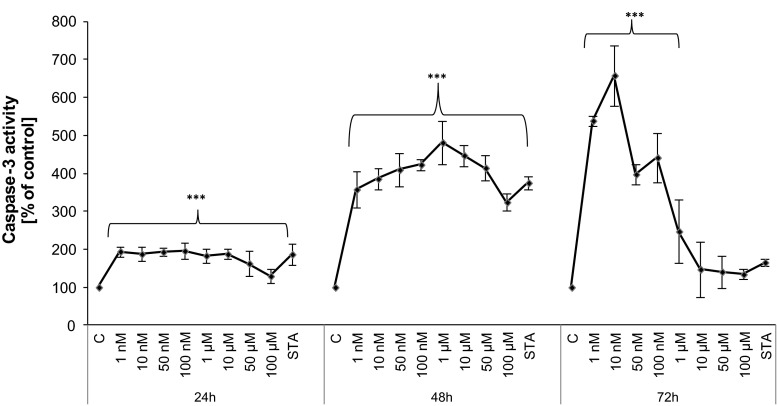
Time- and dose-dependent effect of increasing concentrations of TBBPA on apoptosis in JEG-3 cells as determined by caspase-3 activity. Cells treated with 1 nM of staurosporine (STA) were used as a positive control. Each point represents the mean ± SEM of three independent experiments, each of which consisted of ten replicates per treatment group. Data indicated with ***p < 0.001 reflects statistically significant differences between control and experimental groups

**Fig. 3:**
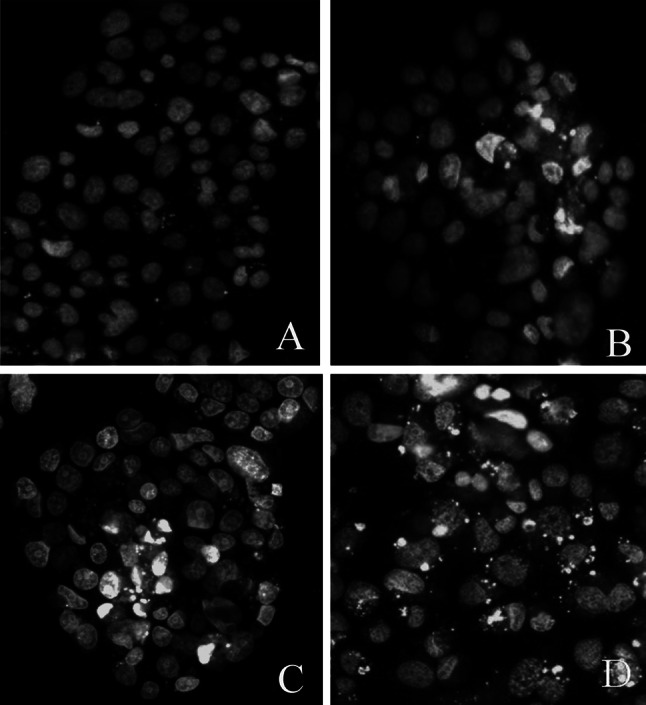
**a**–**d** The effect of TBBPA (10 nM, 100 nM, and 10 µM) on Hoechst 33342 staining in JEG-3 cell cultures, examined 48-h post-treatment. **a** Control cells, **b** 10 nM TBBPA, **c** 100 nM TBBPA, **d** 10 µM TBBPA. No typical apoptotic cells with light-colored cytoplasm were observed in control group (**a**). Cells with bright fragmented nuclei showing condensed chromatin were identified as undergoing apoptosis (**b**–**d**)

TBBPA at all of the tested doses significantly increased caspase-3 activity compared with that of the vehicle control (Fig. 2). The caspase-3 activity was dose-dependent up to 10 and 1 µM of TBBPA for 24 and 48 h, respectively. At these concentrations, the average increase in caspase-3 activity was 1.8- to 2.5-fold after 48 h compared with that observed after 24 h. After 72 h exposure to TBBPA, caspase-3 activity reached the level compared to that of the control cells, suggesting that the final phase of apoptosis occurs upon damage to the cell membrane. The apoptotic action of TBBPA was also confirmed by Hoechst 33342 staining (Fig. 3a–d). Morphological changes, as indicated by bright blue fragmented nuclei, nuclear shrinkage, and subsequent chromatin condensation into the periphery of the nuclei, were identified as apoptotic bodies. The number of apoptotic body increased in a dose-dependent manner.

### β-hCG secretion

**Fig. 4:**
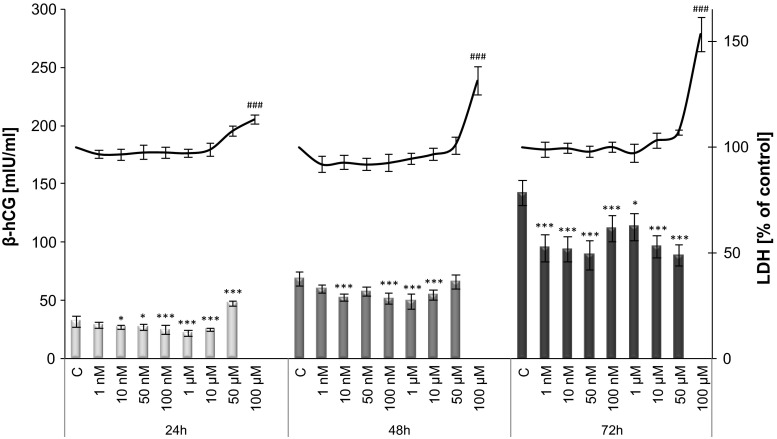
Time- and dose-dependent effect of increasing concentrations of TBBPA on JEG-3 cells viability (*line graph*) and β-hCG secretion (*bar graph*). Each point represents the mean ± SEM of three independent experiments, each of which consisted of ten replicates per treatment group. Data indicated with *p < 0.05; ***p < 0.001 (β-hCG secretion) and ^###^ *p* < 0.001 (LDH release) reflects statistically significant differences between control and experimental groups

TBBPA at nanomolar and micromolar concentrations significantly decreased β-hCG release by JEG-3 cells at all culture time points (24–72 h) (Fig. 4). TBBPA-induced decrease in β-hCG secretion ranged from 15 to 37 % compared to control (vehicle-treated) cells. The highest TBBPA dose (100 μM) showed a strong cytotoxic effect as demonstrated by an increase in LDH release and a low undetectable medium level of β-hCG.

### Effect of the PPARγ agonist GW1929 or antagonist GW9662 on the TBBPA-induced decrease of β-hCG secretion

**Fig. 5:**
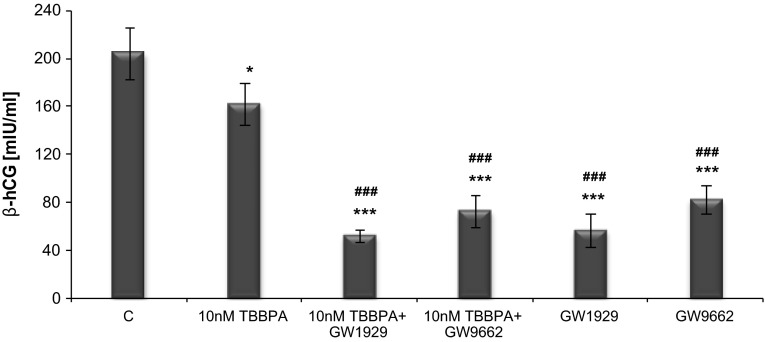
The effect of TBBPA (10 nM) in the presence of PPARγ agonist GW1929 and antagonist GW9662 on β-hCG secretion in JEG-3 cells after 48 h of exposure. Each point represents the mean ± SEM of two independent experiments, each of which consisted of ten replicates per treatment group. Data indicated with ***p < 0.001 reflects statistically significant differences relative to the control. Data indicated with ^###^ *p* < 0.001 reflects statistically significant differences relative to TBBPA

48 h of treatment with TBBPA at concentrations of 10 nM caused significant decreases in the β-hCG production of approximately 31 %, compared with that of the vehicle control (Fig. 5). However, after this time, co-treatment with antagonist did not restore the TBBPA-mediated decrease in the β-hCG level. In contrast, co-treatment with antagonist and TBBPA led to a 1.9-fold decrease in β-hCG production compared with that of TBBPA treatment alone. Interestingly, treatment with antagonist alone caused a 1.5-fold decrease in β-hCG secretion compared with that found upon TBBPA treatment. Treatment with agonist also reduced β-hCG secretion, but the effect was much greater and resulted in a significant decrease that was approximately 2.3-fold higher than that of the TBBPA treatment. Agonist as well as TBBPA reduced β-hCG release, but both exhibited synergy with co-treatment.

### Effect of TBBPA or/and GW1929 or GW9662 on the expression of PPARγ in JEG-3 cells

**Fig. 6:**
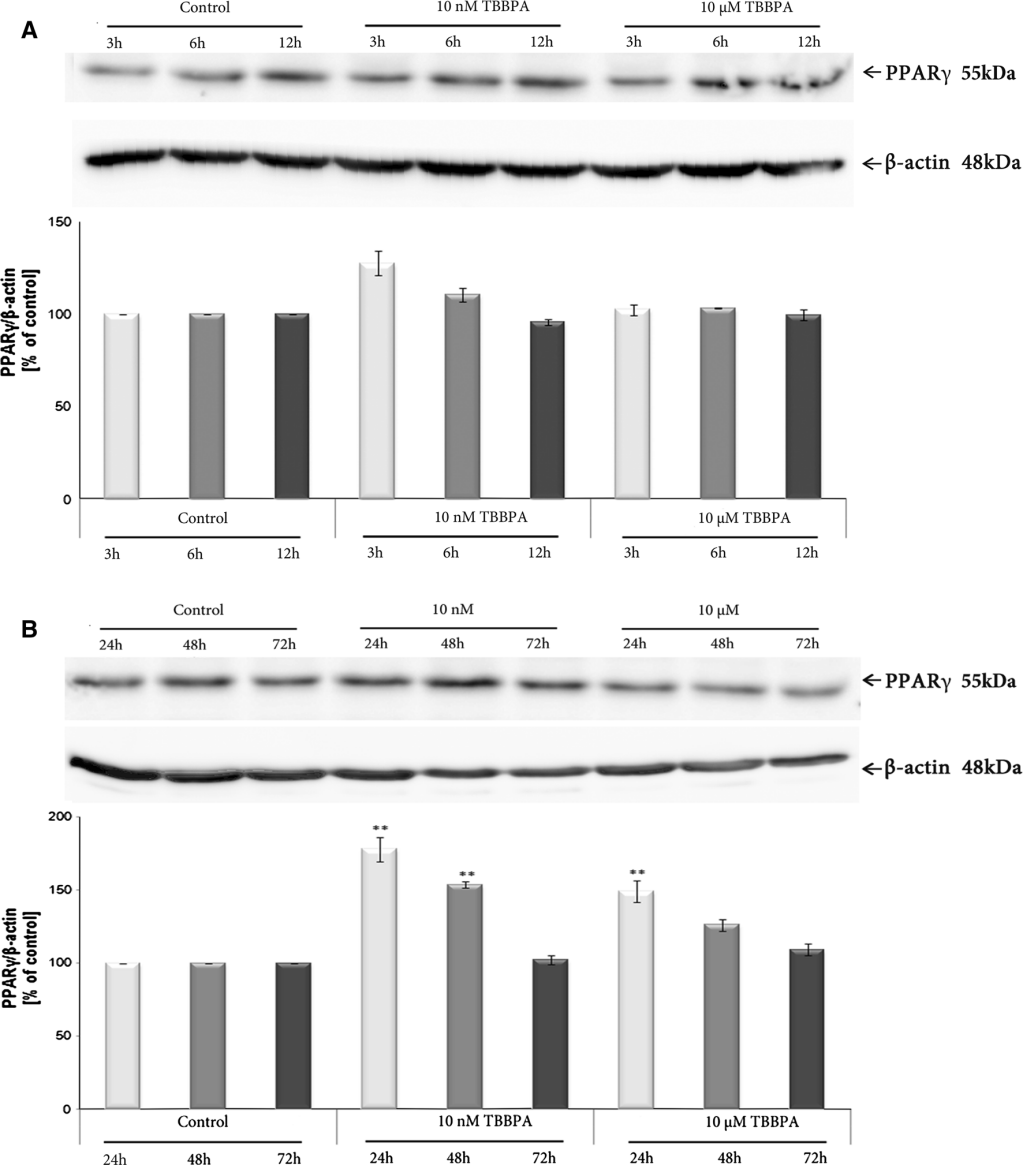
**a**, **b** The *upper panel* shows a representative Western blot of PPARγ protein levels in JEG-3 cells treated with TBBPA (10 nM and 10 µM) for 3, 6, and 12 h (**a**) and 24, 48, and 72 h (**b**). The *lower panel* shows pooled data of three independent experiments. The Western blot membrane was cut into strips and probed with an anti-β-actin antibody to control the amounts for protein loading. PPARγ bands were quantified by densitometry. The results are shown as the percentage of PPARγ protein relative to the control. Data indicated with **p < 0.01 reflects statistically significant differences relative to the control

**Fig. 7:**
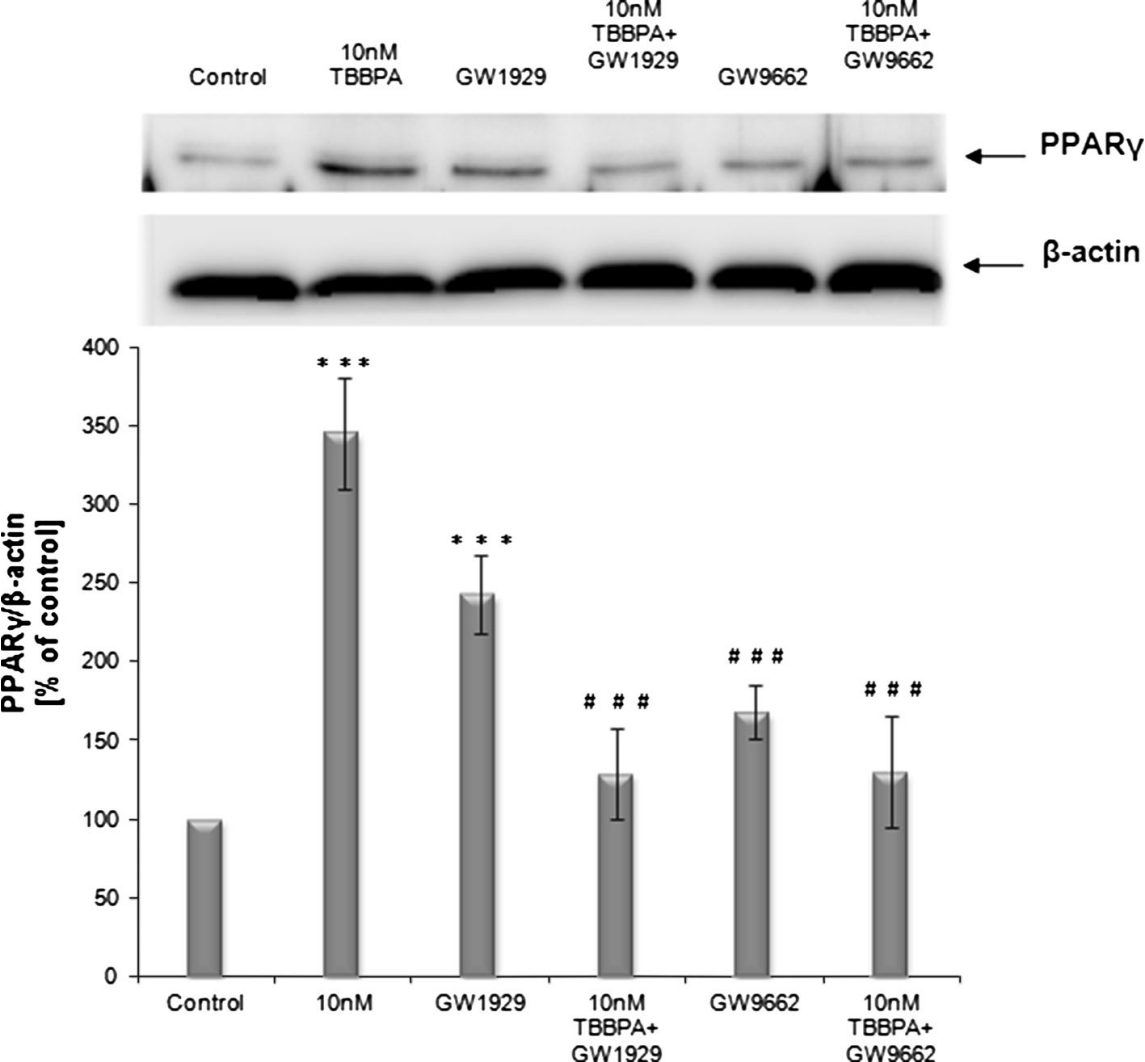
The *upper panel* shows a representative Western blot of PPARγ protein levels in JEG-3 cells treated with TBBPA (10 nM), GW1929 (10 µM), co-treated with TBBPA (10 nM) and GW1929 (10 µM), GW9662 (10 µM), co-treated with TBBPA (10 nM) and GW9662 (10 µM) for 48 h. The *lower panel* shows pooled data of three independent experiments. The Western blot membrane was cut into strips and probed with an anti-β-actin antibody to control the amounts for protein loading. PPARγ bands were quantified by densitometry. The results are shown as the percentage of PPARγ protein relative to the control. Data indicated with ***p < 0.001 reflects statistically significant differences relative to the control. Data indicated with ^###^ *p* < 0.001 reflects statistically significant differences relative to TBBPA

The JEG-3 cell line that was used in this study was found to express the PPARγ protein, in agreement with previous studies [32]. Immunoblot analyses showed that compared with the control cells, TBBPA at concentrations of 10 nM and 10 µM increased the expression of the PPARγ protein in JEG-3 cells after 3–72 h of exposure (Fig. 6a, b). PPARγ protein expression increased in a time-dependent manner until 48 h and then slightly decreased at 72 h but remained above the control level. For a subsequent analysis, we measured PPARγ protein expression after TBBPA treatment at a concentration of 10 nM and a single time point of 48 h. To better understand the relationship between PPARγ protein expression and TBBPA treatment, we used a PPARγ agonist (GW1929) or antagonist (GW9662). Immunoblot analyses demonstrated that 10 nM TBBPA caused a 3.5-fold increase in the expression of the PPARγ protein compared with the control level (Fig. 7). Interestingly, treatment with agonist alone also caused a significant increase in PPARγ protein expression compared with that of the solvent control. Co-treatment with agonist or antagonist caused a modest but insignificant increase in the PPARγ protein expression.

## Discussion

TBBPA is widely used as a flame retardant, but it also has a well-documented endocrine-related biological activity. In particular, higher concentrations of TBBPA in infants compared with their mothers [23] generate great concern because there is a possibility that TBBPA might affect placental function. In this study, we used the human choriocarcinoma-derived placental JEG-3 cell line, which is a reliable model in studies of placental function. This cell line possesses many biological and biochemical characteristics of syncytiotrophoblasts [32] and produces placental hormones [33, 34]. This study showed, for the first time that TBBPA treatment disturbed the synthesis of progesterone by placental cells, the impact of TBBPA on the synthesis of progesterone by placental cells. Our results indicate that TBBPA treatment affected progesterone secretion at all time points compared with the control. An increase in progesterone secretion was significant after 24 h of treatment with TBBPA in the micromolar range and also after 48 h of treatment with TBBPA in the nanomolar range. The results of our previous studies demonstrated that TBBPA also exerted a marked stimulatory effect on estradiol secretion by JEG-3 cells [29]. Progesterone together with estradiol keeps the placenta functioning properly. Estradiol regulates the uptake of LDL particles, which is the first and rate-limiting step in progesterone synthesis [35]. These hormones mutually regulate each other in placenta steroidogenesis, which was confirmed in two independent experiments. Wunsch et al. [36] showed that the antiestrogen MER-25 and the aromatase inhibitor 4-OHA reduced progesterone production in primary cultures of placental cells from pregnant woman at term. Moreover, the marked reduction in progesterone formation was reversed by the addition of estradiol. These results agree with those reported by Shanker et al. [37] who also observed a regulatory role of estradiol in progesterone synthesis in primary cultures of first trimester human placental cells. Our findings, as well as those of other investigators, indicate that one mechanism of TBBPA-mediated increase in progesterone secretion could be associated, at least in part, with the ability of TBBPA to increase the estradiol level. Furthermore, estradiol and progesterone increase CYP11A1 mRNA in cultured human syncytiotrophoblasts, which may suggest a positive feedback mechanism from placental steroids [38, 39]. CYP11A1 catalyzed the side-chain cleavage of cholesterol, which is rate-limiting in the synthesis of progesterone by the human placenta [40]. Interestingly, Dankers et al. [41] also reported that TBBPA moderately induced steroidogenic cytochrome P450scc (CYP11A1) gene expression in a murine Leydig (Ma-10) cell line. CYP11A1 not only seems to be the key regulator of steroidogenesis, but it may also be involved in the induction of apoptosis [42]. In the present investigation, the relationship between the CYP11A1 gene and the apoptosis of trophoblast cells was not explored, but He et al. [43] investigated this issue. These authors showed that the overexpression of CYP11A1 in the trophoblast cell line HTR-8/SVneo induced cell apoptosis through the activation of caspase-3 expression. Our data revealed the proapoptotic effects of TBBPA in JEG-3 cells via the induction of a prominent increase in caspase-3 activity. This stimulatory effect was observed after 24 and 48 h of treatment with TBBPA at doses of up to 10 and 1 µM, respectively, compared with that of the vehicle control. A 72-h exposure to TBBPA resulted in a level of caspase-3 activity approximate to that of control cells, suggesting that the final phase of apoptosis occurs following damage to the cell membrane. The apoptotic action of TBBPA was also confirmed by Hoechst 33342 staining. Although additional studies are required to determine the mechanism of TBBPA action, our findings, as well as those of He et al., provide valuable input into the molecular mechanism of endocrine disruption in human placental cells. The results of our study describing the effect of TBBPA on the activation of caspase-3 can only be compared with results from studies using different biological models. So far, TBBPA at micromolar concentrations has been shown to induce apoptosis in mouse primary neuronal cell cultures [44], rat pheochromocytoma PC-12 [45] and SH-SY5Y human neuroblastoma cells [46], and in Sertoli cells [47]. In these studies, the proapoptotic action of TBBPA occurred only at micromolar concentrations, whereas even nanomolar concentrations were effective in the studies presented above.

The data presented herein also demonstrate the adverse effect of TBBPA on β-hCG secretion in JEG-3 cells, resulting in a significantly decreased hormone level after both short-term (24 and 48 h) and long-term (72 h) exposure. An inhibitory effect was observed at a wide range of TBBPA concentrations (1 nM–10 µM). TBBPA at this range of concentrations did not affect JEG-3 cell viability, whereas the level of β-hCG decreased as the time of exposure increased. These results suggest that an altered hormone level is associated with the effect of TBBPA on β-hCG secretion. To the best of our knowledge, there have been no reports on the action of TBBPA on β-hCG synthesis in human placental cells. There are very few data available regarding the modulation of β-hCG synthesis in human placental cells by trialkyltin [48], DDT and its metabolite [49] or TCDD [50]. These studies, however, do not explain the molecular mechanism that is responsible for the adverse effects on β-hCG secretion. Wójtowicz et al. [49] suggested that a possible mechanism of hCG inhibition may involve the elevation of the progesterone level. A dose-dependent progesterone inhibitory action on hCG secretion or/and production was found in human term placental explant culture [51, 52]. This hypothesis could agree with our data, demonstrating a decreased β-hCG level and increased progesterone level, but additional experimental evidence is required.

It is clearly established that hCG gene expression is regulated by PPARγ [53]. It also has been postulated that PPARγ stimulates hCG synthesis and trophoblast differentiation [54, 55]. The results in our study showed that impairment in β-hCG secretion is accompanied by increased levels of the PPARγ protein. An immunoblot analysis demonstrated that after TBBPA treatment at 10 nM and 10 µM, PPARγ protein expression increased in a time-dependent manner until 48 h and then slightly decreased at 72 h but remained above the control level. Therefore, we hypothesized that in our study, PPARγ is involved in TBBPA-mediated decrease in β-hCG secretion. PPARγ involvement in the mechanism of TBBPA action was demonstrated in NIH3T3-L1 preadipocytes and in mouse neocortical cells. It has recently been reported that PPARs are targets for TBBPA action [28, 44].

The JEG-3 cell line that was used in our study was found to express the PPARγ protein, in agreement with previous studies [32]. Our data are the only to show the up-regulation of PPARγ protein expression after TBBPA exposure in human placental cells. When JEG-3 cells were treated with 10 nM of TBBPA, PPARγ protein expression increased almost 3.5-fold compared with that of control cells. It is noteworthy that TBBPA at 10 nM had a more profound effect on PPARγ expression than did a specific agonist alone, which caused only a 2.5-fold increase. Co-treatment of TBBPA with PPARγ antagonist restored TBBPA-mediated increase in PPARγ protein level. A similar results were observed for co-treatment of TBBPA with PPARγ agonist. It is possible that both TBBPA and GW1929 competed for the same binding site on the receptor.

To explain the relationship between PPARγ and β-hCG secretion, the effect of selective PPARγ antagonist (GW9662) was examined. Although co-treatment with GW9662 reversed the TBBPA-mediated increase in PPARγ protein expression and restored it to the control level, it did not reverse the effect on β-hCG secretion. According to β-hCG secretion, co-treatment with GW9662 potentiated an inhibitory effect of TBBPA, leading to a decrease in β-hCG secretion to a level much lower than that found in TBBPA treatment alone. Moreover, the effect of GW9662 and TBBPA on β-hCG secretion was similar to that of GW9662 alone. Our data also showed that treatment with GW9662 alone caused an increase in the PPARγ protein level compared with that of the solvent control. Similarly, Levytska et al. [56] documented an increase in the PPARγ protein level after treatment with T0070907, an antagonist of this receptor, in BeWo choriocarcinoma cells. Moreover, our results agree with those reported by Levytska et al., who found that blocking PPARγ activity with T0070907 resulted in a significant downregulation in β-hCG secretion by BeWo cells. It may suggest that a negative-feedback mechanism regulates the self-expression of PPARγ protein.

The data presented herein demonstrated that although TBBPA exerts some of its action via the activation of PPARγ, there are clearly alternate mechanisms that are involved in its biological actions, particularly in a PPARγ-independent manner. Additional experiments are required to verify this hypothesis. Considering the important role of PPARγ in placenta development and our findings that an alteration in PPARγ protein expression was accompanied by decreased β-hCG levels at each time point, further research is required to elucidate a possible role of PPARγ in TBBPA-mediated endocrine disrupting effects. The available literature provides some evidence that an alteration in the PPARγ protein expression pattern may be involved in different pathologies of the human placenta. Holdsworth-Carson et al. [57] examined placentas in pregnancies that were complicated with preeclampsia and intrauterine growth restriction and demonstrated significant increases in PPARγ expression. Moreover, Fournier et al. [26] suggested that the over-activation of the PPARγ receptor at the maternal-fetal interface could impair implantation and placentation, thereby affecting embryonic development.

In summary, our results demonstrate for the first time that TBBPA is able to alter the progesterone and β-hCG production of JEG-3 cells and revealed proapoptotic effects via the induction of a prominent increase in caspase-3 activity. We also assessed the ability of TBBPA to alter PPARγ protein expression, which was accompanied by decreased β-hCG levels. We speculate that TBBPA exerts its endocrine disrupting properties through complex interactions at the receptor level and could directly or indirectly affect the regulation of β-hCG gene expression in trophoblast, but a complete understanding the mechanism of TBBPA action in placental cells will require further experiments. However, the results are particularly interesting because TBBPA affects placental cells at doses that are equal to the exposure level in a general population.
